# Does Trophectoderm Mitochondrial DNA Content Affect Embryo Developmental and Implantation Potential?

**DOI:** 10.3390/ijms23115976

**Published:** 2022-05-26

**Authors:** Krzysztof Lukaszuk, Amira Podolak

**Affiliations:** 1 Invicta Research and Development Center, 81-740 Sopot, Poland; luka@gumed.edu.pl; 2 Department of Obstetrics and Gynecological Nursing, Faculty of Health Sciences, Medical University of Gdansk, 80-210 Gdansk, Poland

**Keywords:** in vitro fertilization (IVF), preimplantation genetic testing (PGT), embryo, embryo selection, embryo viability, mitochondria, mitochondrial DNA (mtDNA), mitochondrial score (Ms)

## Abstract

A retrospective case control study was undertaken at the molecular biology department of a private center for reproductive medicine in order to determine whether any correlation exists between the mitochondrial DNA (mtDNA) content of trophectoderm and embryo developmental potential. A total of 275 couples underwent IVF treatment, producing a total of 716 embryos. The trophectoderm was biopsied from each embryo at the blastocyst stage (day 5 or day 6 post-fertilization) subjected to low-pass next-generation sequencing (NGS), for the purpose of detecting aneuploidy. For each sample, the number of mtDNA reads obtained after analysis using NGS was divided by the number of reads attributable to the nuclear genome. The mtDNA copy number was found to be higher in aneuploid embryos than in those that were euploid (mean mtDNA ratio ± SD: 1.13 ± 1.37 versus 1.45 ± 1.78, *p* = 0.02) and in day 5 biopsies compared to day 6 biopsies (1.41 ± 1.66 vs. 1.19 ± 1.27, *p* = 0.001), whereas no statistically significant differences in mtDNA content were seen in relation to embryo morphology (1.58 ± 2.44 vs. 2.19 ± 2.89, *p* = 0.12), genetic sex (1.27 ± 1.29 vs. 1.27 ± 1.18, *p* = 0.99), maternal age (1.31 ± 1.41 vs. 1.33 ± 1.29, *p* = 0.43), or its ability to implant (1.14 ± 0.88 vs. 1.21 ± 1.16, *p* = 0.39). mtDNA has small potential to serve as an additional, independent biomarker for embryo selection.

## 1. Introduction

The function of mitochondria in early human development has become an important subject in a number of basic and clinical studies over the last 20 years [1,2,3,4,5,6,7,8,9,10,11,12].

Mitochondria take part in several vital processes including cellular energy production, cell signaling pathways, the regulation of steroidogenesis, and apoptosis. Indeed, the vast majority of cellular ATP is generated in the mitochondrion through the process of oxidative phosphorylation (OXPHOS), which takes place in the electron transport chain [13]. The electron transport chain is the only mammalian cellular apparatus that has its proteins encoded by two distinct genomes, the chromosomal genome and the exclusively maternally inherited mitochondrial genome [14].

The mtDNA is a double-stranded 16.6 kb genome that encodes several subunits of the electron transport chain, making it directly involved in cellular oxidative phosphorylation. Mitochondrial DNA contains 37 genes, all of which are essential for normal mitochondrial function. Thirteen encoded proteins are involved in oxidative phosphorylation [15].

The way mitochondrial systems have evolved in animals and humans can have profound effects on healthy human reproduction. The mtDNA copy number is important for successful fertilization, embryogenesis, and implantation [16]. It was indicated that oocytes with an insufficient mtDNA amount are prevented from being fertilized [17]. Preimplantation development is an energy-demanding process. Each cell division requires adequate energy levels. The mtDNA of the embryo is maternally inherited; for this reason, the DNA content of oocyte mitochondria determines the energy metabolism of the early embryo [18,19]. Improving the ability to select the best embryo with the greatest chance of implantation and live birth is an important goal of reproductive medicine. Currently, the most common parameter for evaluating the quality of the embryo is still morphology. However, even embryos of the highest blastocyst grade may still fail to implant or may result in a miscarriage. Preimplantation genetic testing for aneuploidies (PGT-A) is an additional useful tool for selecting the best normal embryo [20,21,22]. The current PGT-A diagnostic method—next-generation sequencing (NGS) —as well as the embryo chromosomal status, allows additional information to be obtained regarding the amount of mtDNA.

It has been suggested that elevated levels of mtDNA at the blastocyst stage are associated with aneuploidy and decreased implantation potential for euploid human embryos, and therefore the levels of this genome may reflect embryo viability [23,24,25,26,27,28,29]. However, a number of experiments and observations performed by several study groups obtained conflicting results, suggesting no influence of embryo mtDNA copy number on reproductive success [30,31,32,33,34,35,36]. Therefore, we decided to analyze the relative amount of the mitochondrial DNA among investigated trophectoderm cells (TE) in comparison to the amount of genomic DNA (nDNA). For each sample, the number of mtDNA reads obtained after NGS analysis was divided by the number of reads of nDNA. The mtDNA/nDNA ratio was classified as the mitochondrial score (Ms) and used as an indicator of the mitochondrial copy number per cell.

The primary aim of this retrospective study was to evaluate the relationship between mtDNA content of trophectoderm cells at the blastocyst stage of development with embryos’ ploidy status and implantation potential. Additionally, we decided to examine the link between mtDNA levels and embryo morphology and genetic sex, and maternal age.

## 2. Results

### 2.1. Assocation between mtDNA Content and Embryo Chromosomal Status

Of the 716 examined embryos corresponding to 275 patients undergoing PGT-A, 292 (40.8%) were chromosomally normal and 424 (59.2%) were determined as aneuploid. Overall, 209 euploid blastocysts underwent uterine transfer.

Blastocysts with chromosome errors show higher relative mtDNA amounts compared to euploid embryos (*p* = 0.02) (Table 1).

The distribution of the sex chromosomes in the study group of embryos was as follows: 324 (45.3%) male blastocysts and 392 (54.7%) female blastocysts. The embryo Ms was not related to the sex chromosome distribution (*p* = 0.99) (Table 1). There was also no association between Ms and sex chromosome distribution in euploid embryos (*p* = 0.82) (Table 1).

### 2.2. Association between Maternal Age and Embryo mtDNA Levels

The average age of the study group was 36.7 ± 4.2 (range 22–47 years). The analysis of mtDNA quantity was performed in two age groups: 375 embryos from younger women (average age 33.6 years, range 22–36 years) and 341 embryos from older women (average age 40.1 years, range 37–47 years). Embryo Ms was not related to the age of the patients (*p* = 0.43) (Table 1). We did not observe the effect of female age on mtDNA quantity in euploid embryos (*p* = 0.47) (Table 1). The average age of women with the euploid blastocysts was 36.2 ± 4.1 (range 27–45 years).

### 2.3. MtDNA Content versus Embryo Quality and Viability

We also investigated the association of mtDNA content with embryo quality and viability at the blastocyst stage of development. The embryo quality was defined as good or poor. Good quality included blastocysts with trophectoderm and inner cell mass (ICM) score 1 or 2 according to the criteria described by the Istanbul Consensus Criteria [37]. The poor quality referred to a score 3 of TE or ICM. We did not observe statistically significant differences in mtDNA or quality of blastocysts. Nevertheless, we observed a trend toward an increased mtDNA quantity in poorer quality blastocysts (*p* = 0.12). Moreover, the embryo Ms was also not related to blastocyst quality (*p* = 0.14) in euploid embryos (Table 1). However, for us, another important aspect in assessing the quality of embryos was the rate of blastocyst development. During IVF cycles, the embryonic cohort is asynchronous in development and TE biopsy can equally be performed on day 5 or 6 post-fertilization and on blastocysts of different morphological quality. We analyzed the mtDNA content in blastocysts biopsied on day 5 (n = 421) and day 6 (n = 295) of culture. Day 5 blastocysts had higher relative mtDNA amounts compared to day 6 (*p* = 0.001) (Table 1).

The day of biopsy proved to be statistically significantly associated with the mtDNA levels (*p* = 0.002) also in euploid embryos (Table 1). Moreover, we verified the association between mtDNA content and the day of biopsy in implanted embryos. We observed statistically significantly (*p* = 0.03) decreased levels of mtDNA at day 6 biopsied implanted embryos (n = 23) compared to day 5 biopsy (n = 53) (Table 1).

### 2.4. The Effect of mtDNA Content on Embryo Implantation Potential

The implantation potential of euploid blastocysts was analyzed based on 209 transferred embryos in single embryo transfer (SET) with or without implantation or double embryo transfer (DET) that either led to dizygotic twins or no implantation. Another 83 euploid embryos were frozen as surplus embryos. We did not observe statistically significant differences in mtDNA and clinical outcome (*p* = 0.39). Nevertheless, we noted a trend toward decreased mtDNA quantity in implanted embryos (Table 1).

### 2.5. Mitochondrial Presence

**Figure 1:**
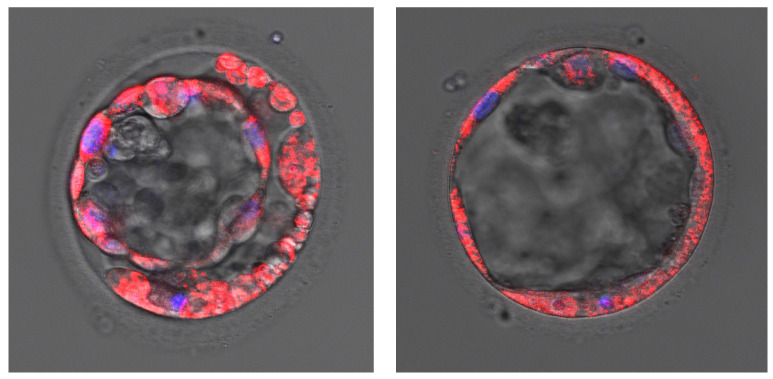
Aneuploid blastocysts. Mitochondria are stained red (MitoTracker Red CMXRos) and nuclei are stained blue (DAPI).

**Figure 2:**
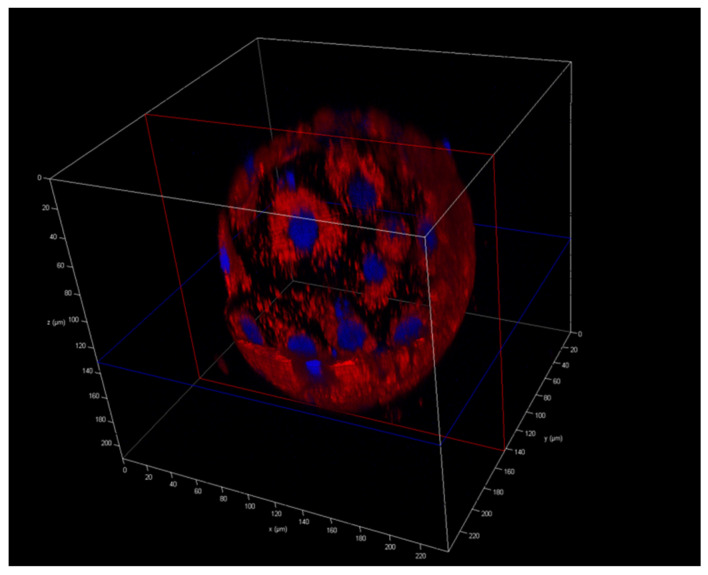
Aneuploid blastocyst in 3D cross-section. Mitochondria are stained red and nuclei are stained blue.

From a biological standpoint, the distribution of the mtDNA between different cells of the trophectoderm and inner cell mass during progression to the blastocyst stage is unknown. In our study, we analyzed the mtDNA level in TE. We decided to stain mitochondria in an aneuploid blastocyst (Figure 1 and Figure 2) following PGT-A NGS results. We observed different distributions of mitochondria in embryos.

## 3. Discussion

The development of assisted reproductive technology is focused on achieving a higher rate of live births [38]. The application of new methods for the selection of the best embryo are still underway. The gold standard to achieve the expected success seems to be preimplantation genetic testing for aneuploidies. It became a priority to obtain an euploid embryo with good morphology—the perfect embryo. However, it soon became apparent that this does not guarantee of a successful pregnancy. New indicators are constantly being sought to assess and select the ideal embryo.

From a biological point of view, preimplantation development is an energy-demanding process. Each cell division requires adequate energy levels. The mtDNA of the embryo is maternally inherited, with the oocyte harboring around 500,000 copies [18,19]. The amount of mtDNA remains stable throughout the cleavage stages with mtDNA replication beginning at the blastocyst stage or post-implantation [39,40]. Mitochondrial DNA accumulates in the mature oocyte and is expanded from 100 copies per primordial germ cell to 1,000,000 copies at the metaphase II (MII) stage [41]. The total amount of oocyte mtDNA must be split among cells during embryo development. The mtDNA amount positively correlates with fertilization and embryo viability, which may suggest that it could be a good biomarker of oocyte quality [17,42,43]. For that reason, in recent years, several research teams have paid attention to the implication of embryo mtDNA level on reproductive outcome. However, the obtained results are highly conflicting and the utility of mitochondrial score as an embryo selection marker is still unknown [23,24,25,26,27,29,30,31,32,33,34,35,36,44,45].

We have demonstrated higher mtDNA copy number in aneuploid embryos compared to euploid ones. These data confirmed the results of most other studies as well as our previous study conducted with blastomeres biopsied from cleavage-stage embryos [23,30,35,46,47,48]. The number of mtDNA copies can affect all vital processes that take place in cells, but mostly the specialized processes that require most of the energy for particular functions. This could also influence the functioning of the gametes and disturb reproductive possibilities. Elevated mitochondrial DNA copy number is observed in aneuploid embryos, thus indicating that this parameter assessment could be an additional tool for the selection of embryos for the transfer of small value. Moreover, fluorescence staining demonstrated divergent distribution of mitochondria among embryos and confirmed that this process is variable and dynamic and should be further evaluated to understand the biology of the preimplantation embryo.

In the context of embryo quality and viability, no association between mtDNA levels and embryo morphology was found. However, mtDNA content was found to correlate with embryo development rate. During IVF cycles, the embryonic cohort is asynchronous in development and TE biopsy can either be performed on day 5 or day 6 post-fertilization and on blastocysts of different morphological quality. It is still unknown whether blastocyst morphology and developmental rate are related to the embryo chromosomal constitution. The study of Sesh Kamal Sunkara et al. shows that slower-developing blastocysts cryopreserved on day 6 but at the same stage of development as those developing to the blastocyst stage on day 5 do not have similar chromosomal status. Euploid embryos tend to show faster progression to the most advanced expansion stages when compared with aneuploid embryos [49]. Four years later, Capalbo et al. performed a multicenter retrospective observational study including 956 blastocysts. They assessed correlation between standard blastocysts’ morphology, euploidy, and implantation [50]. This study suggests that the commonly used parameters of blastocyst evaluation are not good enough indicators to improve the selection among euploid embryos. Accordingly, all morphologically poor and slower-growing expanded blastocysts should be biopsied and similarly considered for transfer. We analyzed the mtDNA score in blastocysts biopsied on day 5 and day 6 of culture. Day 5 blastocysts show higher relative mtDNA amounts compared to day 6. We observed the same relationship in euploid embryos. These results are consistent with findings of other research teams [29,33,44,45]. As day 5 blastocysts are considered to provide higher implantation rates [51,52,53,54], higher mtDNA content may be associated with embryo developmental potential. Additionally, we confirmed the association between Ms and the day of TE biopsy in successfully implanted embryos.

However, there was no statistically significant difference in mtDNA score between embryos that implanted and those that did not, suggesting no impact of trophectoderm mtDNA content on embryo implantation competence. These findings are consistent with our previous study performed with cleavage-stage embryos [35].

The possibility of using mtDNA score as an embryo selection marker to improve implantation rates was indicated for the first time in 2015 by Fragouli et al. [23]. The established thresholds were also verified in the study of Ravichandran et al. [27]. However, 100% negative predictive value of mtDNA assessment was concluded from 33 embryos containing elevated levels of mtDNA that failed to produce pregnancy [27]. Such a sample size seems far too small to draw any conclusion. The correlation of elevated quantity of mtDNA copy number with a lower embryo viability was subsequently demonstrated by several investigators [25,26,29]. However, even though Wang et al. [29] found a statistically significant difference in mtDNA content between implanted and nonimplanted embryos, they considered mtDNA score as independent embryo selection marker by receiver operating characteristic (ROC) curve analysis and found a low predictive value of this parameter in embryo implantation and live birth outcomes. Interestingly, Wu et al. [45] found a positive correlation of mtDNA score value with an embryo’s ability to implant. Furthermore, a number of conducted studies did not find any correlation between these variables. How is it possible that obtained results show such a divergence? First of all, we should consider mtDNA score estimation technique. There are several calculation formulas that may influence the obtained findings in different ways [23,25,34,36,45]. Second, technical approaches may also play a crucial role when taking into consideration that even investigators who used the same formula obtained conflicting results. For instance, Victor et al. [36] found no association of mtDNA score with embryo implantation competence, whereas Wang et al. [29], when using their formula, observed negative correlation between embryos’ mtDNA content and their ability to implant. Similarly, Shang et al. [34] indicated no impact of mtDNA level on implantation potential, whereas Du et al. [26], when using their formula, reported statistically significant differences in the quantity of mtDNA copy number between implanted and non-implanted embryos. The WGA protocol used for the assessment may be one of the possible factors influencing obtained results as it may affect the amplification of mtDNA compared to nDNA [35]. Similar to our previous study, mtDNA after WGA was amplified mostly in two regions—from 65 to 500 (HRV2) and from 9950 to 10270. As it was consistent and reproducible, this allowed us to analyze the relative amounts of mitochondrial DNA without preventing the reliable assessment; however, it does not provide sufficient reliability for more detailed sequence analysis, which must be highlighted as the main limitation of our research.

Moreover, similar to our previous findings and the majority of other conducted studies, we did not find any link between mtDNA levels and maternal age [29,30,32,33,35,36,44,45,46].

As mentioned above, the main limitation of our study was the preferential amplification of two regions of mtDNA. However, the lack of evaluation of pathogenic mtDNA mutations as a factor possibly influencing our results should also be taken into account. However, it must be highlighted that method used is designed to assess the number of chromosomes and mtDNA and such approach affords decreased costs and faster analysis. Furthermore, we have not taken heteroplasmy into consideration as such analysis demands deeper sequencing and, probably, a change in the WGA technique. We believe that further investigation of qualitative aspects of mtDNA possibly influencing reproductive success should be evaluated.

## 4. Materials and Methods

### 4.1. Study Design

This retrospective study examined 716 trophectoderm blastocyst biopsies from 275 patients undergoing PGT-A between January 2014 and December 2015 at a molecular biology department of a private fertility clinic. For the purpose of this study, the sample size was calculated with a confidence level defined as 95%, alpha 0.05, and statistical power set as 80%.

We aimed to assess the impact of mtDNA content on embryos’ developmental and implantation potential at the blastocyst stage of development. In our previous study, we examined the abovementioned parameter in cleavage-stage embryos [35]. In this study, we decided to include the embryos of corresponding timeframe so that the comparison would not be affected by the change in either laboratory protocols or the staff performing PGT-A.

The inclusion criteria were: (1) indications for PGT-A (Table 2), (2) biopsy performed at blastocyst stage of development, (3) patients with a known pregnancy outcome until clinical pregnancy or miscarriage was confirmed. The study excluded patients with intrauterine anomalies. Relevant clinical details, medical history, and pedigree were documented during infertility treatment. All samples were assessed at Invicta Medical Laboratories, Molecular Biology Department. The quantity of mtDNA reads was calculated from BAM files of NGS data and followed by analysis of mtDNA/nDNA ratio.

### 4.2. Stimulation Protocol, Oocyte Retrieval

In our clinic, all women were treated with a long agonist protocol starting from oral contraceptives (OCs) (Ovulastan, Adamed, Czosnow, Poland) from day 2–5 of the cycle. Triptorelin acetate 0.1 mg (Gonapeptyl, Ferring, Saint-Prex, Switzerland) was administered 14 days after the beginning of the OCs. Fourteen days later (seven days after the end of OC administration), urinary gonadotropins (Menopur, Ferring, Saint-Prex, Switzerland) for ovarian stimulation were administered. The dosage administered was dependent on AMH level, and ranged from 150 to 300 IU daily (12). Follicular growth was monitored on stimulation day 8 using transvaginal ultrasound and assays evaluating serum estradiol (E2), progesterone (P), and luteinizing hormone (LH) levels. Oocyte pick-up was performed 36 h after the administration of 5000 IU of hCG (Choragon, Ferring, Saint-Prex, Switzerland).

### 4.3. Embryo Culture, Biopsy, and Vitrification

All examined embryos were cultured in sequential media G1 and G2 (Vitrolife, Västra Frölunda, Sweden) to blastocyst stage under 6% CO_2_ and 5% O_2_ at 37 °C. On day 5 after fertilization, blastocysts were graded according to the Istanbul Consensus Workshop on Embryo Assessment [37]. Trophectoderm blastocyst biopsies were performed only for blastocysts with grade over 211. Slower-developing blastocysts were cultured until day 6 or 7 and, when they reached good morphology (over 211), subjected to trophectoderm biopsy. Approximately 5–7 TE cells located opposite to the inner cell mass were aspirated with a biopsy pipette and dissected with a laser (Hamilton Thorne Inc., Beverly, MA, USA). Biopsied TE cells were washed in phosphate-buffered saline (PBS) (Cell Signaling Technology, Danvers, MA, USA)/polyvinylpyrrolidone (PVP) (Sigma-Aldrich, Saint Louis, MO, USA) and stored at −196 °C for subsequent PGT-A analysis. Blastocysts were vitrified immediately after the biopsy using the Kitazato vitrification kit (Kitazato, Shizuoka, Japan) according to the manufacturer’s protocols.

### 4.4. Whole Genome Amplification (WGA) and Next-Generation Sequencing (NGS)

To perform next-generation sequencing, trophectoderm cells from each embryo were amplified using the PicoPLEX WGA Kit (New England BioLabs Inc., Ipswich, MA, USA) according to the manufacturer’s protocol. The DNA concentration after WGA was determined with Qubit 2.0 Fluorometer and Qubit dsDNA HS Assay (Invitrogen, Waltham, MA, USA). Libraries were prepared with an Ion Xpress Plus Fragment Library Kit (Ion Torrent, Waltham, MA, USA) according to the manufacturer’s protocol for 10–100 μg of genomic DNA. For barcoding, an Ion Xpress Barcode Adapters 1–96 Kit (Ion Torrent, Waltham, MA, USA) was used. The Ion Xpress Equalizer Kit (Ion Torrent, Waltham, MA, USA) was used for library input normalization according to the manufacturer’s instructions. For library enrichment, an Ion PGMTM Template OT2 200 Kit (Ion Torrent, Waltham, MA, USA) was used according to the manufacturer’s protocol. The sequencing was performed using the Ion PGMTM Sequencing 200 Kit v2 (Ion Torrent, Waltham, MA, USA) on an Ion 316 chips (Ion Torrent, Waltham, MA, USA). Up to 32 samples per 316 chip were processed. Ion Torrent Suite Software enabled preliminary analysis, e.g., base calling and read mapping against a human genome reference sequence (hg19). Data were analyzed using the Coverage Analysis (V. 5.12.0.0) plugin in the Torrent Suite V. 5.12.3 (Thermo Scientific, Waltham, MA, USA), providing the percentage of DNA sequence reads mapped to each chromosome. Detection of aneuploidies was performed using sample results compared to baseline values obtained from 85 male and 83 female euploid single-cell samples processed beforehand with an established protocol [55]. Control samples served as a positive control for the entire process. Additionally, a negative control sample was processed to exclude the possibility of contamination. The NGS method was designed by the Molecular Biology Department of Invicta Medical Laboratories and validated using human cell lines.

### 4.5. Determination of Mitochondrial DNA Copy Number

As for our previous study, nuclear DNA (nDNA) was estimated as the sum of reads obtained from analysis of all chromosomes taking into consideration the presence of monosomy or trisomy [35]. Mitochondrial score (Ms) was classified as the ratio of mtDNA/nDNA and used as an indicator of the mitochondrial copy number per cell. The results were multiplied by 1000 for the best estimation.

### 4.6. Blastocyst Warming and Transfer

For the frozen embryo transfer cycle, euploid blastocysts were transferred to each patient. Blastocysts were warmed using the Kitazato thawing kit [56]. After warming, the blastocysts were transferred to G2 medium and cultured for 2–3 h. Blastocysts were transferred after prior endometrial preparation. The luteal phase was supplemented by transvaginal E2 administration (3 × 2 mg, Estrofem, Bagsvaerd, NovoNordisk) starting from day 2 of the cycle. When the endometrium reached >8 mm thickness, transvaginal P (3 × 100 mg Lutinus, Ferring) was administered. E2, P, and hCG hormone levels were verified every 3–4 days after the transfer.

### 4.7. Cell Fluorescence Staining

For the analysis of the mitochondrial position, aneuploid blastocysts were stained by MitoTracker Red CMXRos (Invitrogen, Waltham, MA, USA) and DAPI (Invitrogen, Waltham, MA, USA) with a standard protocol. The specimens were imaged with a confocal laser scanning microscope (Leica TCS SP8X, Wetzlar, Germany) with a 20× lens and oil and analyzed using LAS AF 3.2 software (Leica, Wetzlar, Germany).

### 4.8. Statistical Analysis

The data analysis was performed using the software system STATISTICA, Version 10 (StatSoft Power Solutions, Inc., Tulsa, OK, USA). To estimate the existing differences in mtDNA quantity the obtained data were analyzed for: (1) aneuploidy and presence of euploid blastomeres; (2) successfully implanted embryos and those that failed to implant; (3) embryos of good and poor morphology; (4) embryos with and without an Y chromosome; (5) embryos from older and younger women. The clinical characteristics and outcomes in the investigated groups were compared using nonparametric tests as the obtained values presented significant variation and the distribution was not normal. Pearson chi-square test and the Mann–Whitney U test were applied to compare implantation rates among the Ms groups. A value of *p* < 0.05 was considered statistically significant in all tests.

## 5. Conclusions

The elevated levels of mitochondrial DNA at the blastocyst stage of development are associated with aneuploidy and decreased implantation potential for euploid human embryos; therefore, the levels of this genome may reflect embryo viability. Moreover, day 5 biopsied embryos tended to present higher mtDNA levels compared to those biopsied at day 6, suggesting that mtDNA content may be associated with embryo developmental potential. However, the relationship between embryo mtDNA content and its ability to implant in the uterus turned out to be statistically insignificant, indicating that the mitochondrial score has only a small potential to become an additional, independent marker for embryo selection. Furthermore, the mtDNA/nDNA ratio did not correlate with either maternal age, embryo genetic sex, or embryo quality.

## Figures and Tables

**Table 1:** Mitochondrial score values for all comparison groups.

Variable	Analyzed Group	N (%)	Mean Ms ± SD	*p* Value
Analyzed Embryos				
Ploidy status	Euploid	292 (40.8%)	1.13 ± 1.37	0.02
	Aneuploid	424 (59.2%)	1.45 ± 1.78	
Genetic sex	With chr. Y	324 (45.3%)	1.27 ± 1.29	0.99
	Without chr. Y	392 (54.7%)	1.27 ± 1.18	
Maternal age	<37 years	375 (53.4%)	1.31 ± 1.41	0.43
	≥37 years	341 (47.6%)	1.33 ± 1.29	
Embryo morphology (Istanbul criteria)	Good	639 (89.2%)	1.58 ± 2.44	0.12
	Poor	77 (10.8%)	2.19 ± 2.89	
TE biopsy day	Day 5	421 (58.8%)	1.41 ± 1.66	0.001
	Day 6	295 (41.2%)	1.19 ± 1.27	
Euploid Embryos				
Genetic sex	With chr. Y	127 (43.5%)	1.13 ± 1.47	0.82
	Without chr. Y	165 (56.5%)	1.23 ± 1.31	
Maternal age	<37 years	172 (58.9%)	1.16 ± 0.93	0.47
	≥37 years	120 (41.1%)	1.08 ± 1.05	
Embryo morphology (Istanbul criteria)	Good	264 (90.4%)	1.38 ± 2.12	0.14
	Poor	28 (9.6%)	1.87 ± 2.56	
TE biopsy day	Day 5	187 (64%)	1.21 ± 1.03	0.002
	Day 6	105 (36%)	0.98 ± 0.86	
Transferred Embryos				
Implantation status	Implanted	76 (36.4%)	1.14 ± 0.88	0.39
	Non-implanted	133 (63.6%)	1.21 ± 1.16	
Implanted Embryos				
TE biopsy day	Day 5	53 (69.7%)	1.27 ± 0.93	0.03
	Day 6	23 (30.3%)	0.84 ± 0.71	

**Table 2:** Indications for PGT-A.

Indications for PGT-A
recurrent implantation failure (>=2)
recurrent miscarriage (>=2)
maternal age more than 35
severe male factor
age of man more than 50
fear of chromosomal abnormalities in the child

The ethics committee approvals were obtained for this study (reference number ACTRN12614001037695).

## Data Availability

The data presented in this study are available on request from the corresponding author.
